# Computational and neural signatures of pre and post-sensory expectation bias in inferior temporal cortex

**DOI:** 10.1038/s41598-018-31678-x

**Published:** 2018-09-05

**Authors:** Kyle Dunovan, Mark E. Wheeler

**Affiliations:** 1 Department of Psychology, University of Pittsburgh, Pittsburgh, PA USA; 2 Center for the Neural Basis of Cognition, University of Pittsburgh, Pittsburgh, PA USA; 3 School of Psychology, Georgia Institute of Technology, Atlanta, GA USA

## Abstract

As we gather noisy sensory information from the environment, prior knowledge about the likely cause(s) of sensory input can be leveraged to facilitate perceptual judgments. Here, we investigated the computational and neural manifestation of cued expectations in human subjects as they performed a probabilistic face/house discrimination task in which face and house stimuli were preceded by informative or neutral cues. Drift-diffusion modeling of behavioral data showed that cued expectations biased both the baseline (pre-sensory) and drift-rate (post-sensory) of evidence accumulation. By employing a catch-trial functional MRI design we were able to isolate neural signatures of expectation during pre- and post-sensory stages of decision processing in face- and house-selective areas of inferior temporal cortex (ITC). Cue-evoked timecourses were modulated by cues in a manner consistent with a pre-sensory prediction signal that scaled with probability. Sensory-evoked timecourses resembled a prediction-error signal, greater in magnitude for surprising than expected stimuli. Individual differences in baseline and drift-rate biases showed a clear mapping onto pre- and post-sensory fMRI activity in ITC. These findings highlight the specificity of perceptual expectations and provide new insight into the convergence of top-down and bottom-up signals in ITC and their distinct interactions prior to and during sensory processing.

## Introduction

Prior knowledge plays a pivotal role in shaping perception - placing contextual constraints on the interpretation of sensory inputs to facilitate fast, accurate detection of expected stimuli. Recent evidence suggests that expectations and other forms of top-down information exert bias over the processing of lower level sensory inputs at much earlier stages than previously thought. For instance, functional imaging studies in humans have found that category-selective regions of inferior temporal cortex (ITC) exhibit activation patterns more consistent with the accumulation of decision evidence than with raw sensory representation^1^. Studies of top-down signaling in ITC have generated support for predictive coding (PC) theories of perception, which assume that internal expectations are represented hierarchically, from abstract/categorical representations in frontal cortex down to feature-level stimulus templates in the relevant areas of sensory cortex^2^. These low-level feature predictions not only prime sensory cortex for faster feature detection, but also allow the brain to refine its internal model by propagating sensory prediction errors back up to higher-order regions (e.g., prefrontal cortex). Empirical evidence for PC has largely come from human neuroimaging studies showing that, in relevant parts of sensory cortex, stimulus-evoked activity is weaker when a stimulus is expected compared to when it comes as a surprise^3^. This “prediction error” signal has proven to be highly reliable in perceptual decision making studies across visual^4,5^, auditory^6,7^, tactile^8,9^, and olfactory^10^ domains. Fewer studies, however, have focused on how expectations are represented in sensory cortices prior to sensory input.

Bell *et al*.^11^ found compelling evidence for the emergence of expectations in population-level activity in inferior temporal cortex (ITC) as monkeys learned the relative probability of face and fruit stimuli. Critically, they showed that face expectations were associated with an increase in the firing rate of a particular subset of face-selective neurons. Stimulus-evoked activity, on the other hand, was greater for surprising stimuli – consistent with a prediction error signal. Empirical support for PC in humans is mostly drawn from the observed modulatory effects of expectations on *stimulus-evoked* activity (e.g., the prediction-error) whereas relatively less is known about the preceding manifestation of the prediction itself. Kok *et al*.^12^ found compelling, albeit indirect, evidence for top-down predictions in visual cortex – showing that the omission of an expected stimulus elicited a greater response than actually viewing an expected stimulus. Similar to the findings of Bell *et al*.^11^, Puri *et al*.^13^ showed that cued face expectations were associated with an increase in fusiform activation in human subjects performing a face/house discrimination task. However, counter to a PC account, these authors observed a larger stimulus evoked response for expected than unexpected faces, Puri *et al*., concluded that, in addition, to priming sensory populations, prior expectations also serve to amplify the response to anticipated stimuli. Here we sought to reconcile the findings reported by Puri *et al*.^13^ with the growing body of evidence for PC theories by addressing a design flaw in their original study. Due to the sluggish nature of the blood oxygenation-level dependent (BOLD) signal measured by fMRI, it is possible that the amplified response to expected stimuli observed by Puri *et al*.^13^ was actually the result of carryover activity from the prior prediction signal.

**Figure 1:**
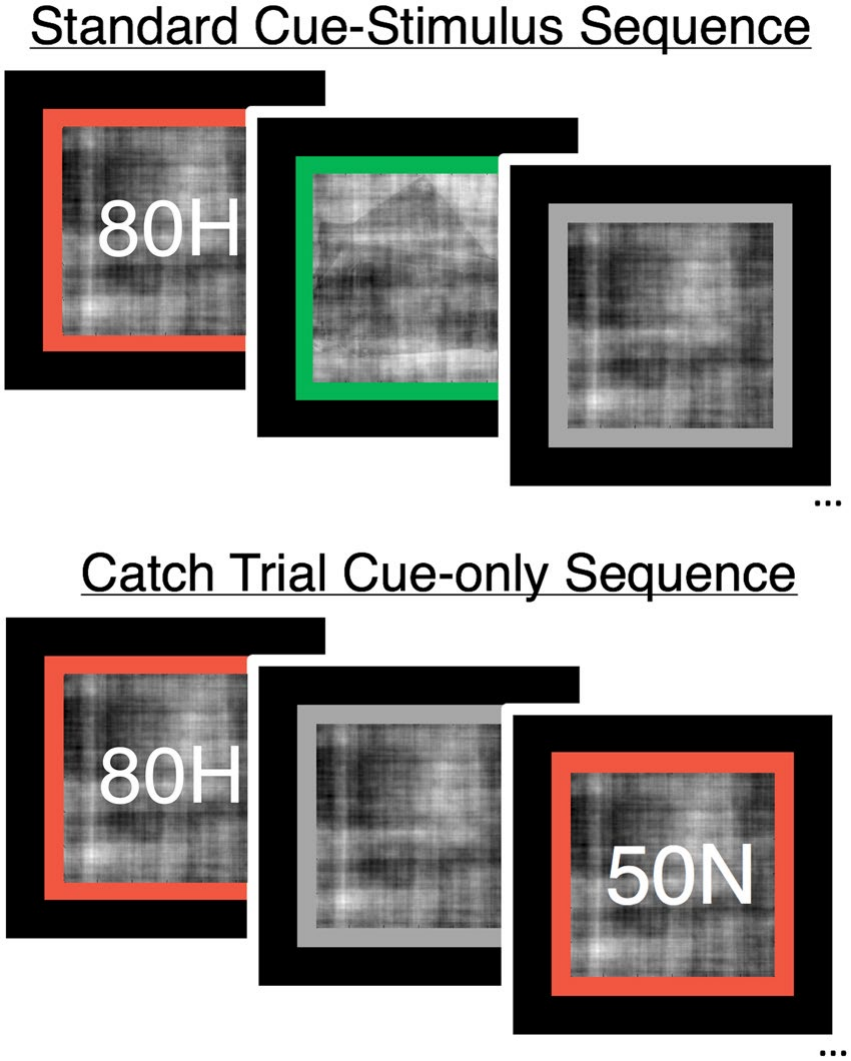
Probabilistic face/house discrimination task. On full trials (top) subjects were shown a cue (letter number combination over noise-only movie; red border) informing them of the prior probability of each stimulus category, followed by a noise-degraded movie of either a face or house stimulus (green border), and finally a jittered rest period (noise-only movie; gray border). On catch trials (bottom), subjects were shown a cue followed immediately by a rest period and were instructed to wait for the next trial.

In addition, we leveraged a well-known model of decision making, the drift-diffusion model (DDM), to formally characterize the behavioral effects of probabilistic cues on pre- and post-sensory decision evidence. The DDM assumes that perceptual decisions are made by accumulating noisy sensory evidence between two decision boundaries, each representing the criterion evidence for committing to a categorical choice. While accumulation-to-bound and PC theories offer different levels of description for perceptual decision phenomena, both frameworks explicitly assume that perceptual decisions arise from a combination of pre- and post-sensory information processing. In a previous study using the same probabilistic face/house task as used in the current study, fits of the DDM to behavioral data showed that cues led to biases in both the baseline evidence (e.g., pre-sensory) and rate of evidence accumulation (e.g., post-sensory). Thus, a further aim of the current study was to replicate our previous modeling results and to determine how biases in parameters of the DDM map onto pre- and post-sensory effects of cued expectations in category-selective ITC. We hypothesized that behaviorally derived starting-point estimates of the DDM should reflect the strength of prediction signals (e.g., with greater starting-point bias in conditions with greater cue-evoked activation); whereas drift-rate estimates should reflect the match between predicted and observed sensory inputs (e.g., with higher drift-rates in conditions with lower sensory-evoked activation). To investigate this hypothesis we collected behavioral and fMRI data from human subjects as they performed a probabilistic face/house discrimination task^14^ (Fig. 1) and compared behaviorally-derived estimates of bias in parameters of the DDM to cue- and sensory-evoked BOLD activity in face- and house-selective regions of ITC.

## Results

### Behavioral Performance

**Figure 2:**
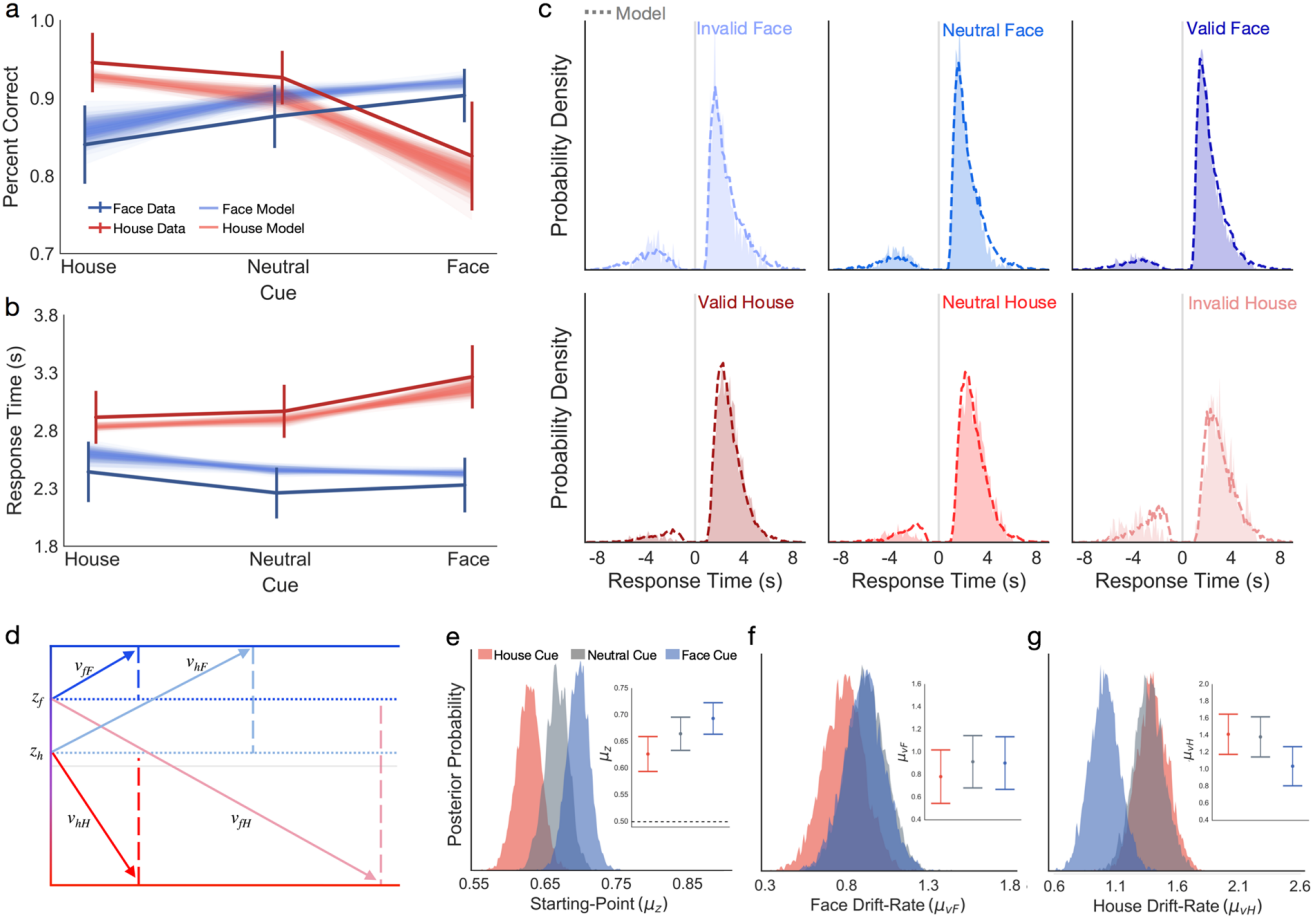
Observed and model-predicted effects of cued expectations on behavior. Average (**a**) accuracy and (**b**) RT (correct only) of face (dark blue line) and house (dark red line) decisions plotted as a function of cue type across subjects (N = 19). Model-predicted accuracy and RT data for each stimulus category are shown as overlays (face: transparent blue; house: transparent red), each line corresponding to predictions from one of 100 simulated datasets. Each simulated dataset was generated using the subject-level parameter estimates of the hierarchical MSM model and contained the same trial count per condition as in the actual experiment to capture predicted estimates of variability. (**c**) Observed (filled) and model-predicted (dashed line) RT distributions for face (blue, top) and house (red, bottom) stimuli, plotted separately for invalid (lighter), neutral, and valid (darker) cued expectations. Error RTs are flipped on the x-axis (left of vertical gray line). Correct RTs are plotted as positive values (right of vertical gray line). (**d**) Schematic showing the effects of valid and invalid prior cues on starting point and drift rate parameters. Posterior probability distributions for (**e**) starting point (*z*_*cue*_), (**f**) face drift-rate (*v*_*F*_), and (**g**) house drift-rate (*v*_*H*_) estimates in the 80F (Face, blue), 50N (Neutral, gray), and 80H (House, red) cue conditions. Error bars in panels (**a**,**b**) show the 95% c.i. around the mean. Inset plots in panels (e–g) show the mean and 95% c.i. of the posterior probability distribution for each of the cues.

Human subjects performed a probabilistic face/house discrimination task in which a preceding cue indicated the prior probability (80% or 50%) of an upcoming stimulus being a face or house (noise-degraded movie; see Online Methods; see Fig. 1). In the analyses, trials were sorted by cue type (80F, 50N, 80H), stimulus (face, house) and accuracy (correct, incorrect). Notably, almost all behavioral effects (Fig. 2) were consistent with our previous results^14^. The interaction between cue and stimulus was significant as a predictor of both accuracy (*F*(1.36, 24.94) = 9.58, *p* < 0.001) and RT (*F*(1.50, 27.02) = 16.84, *p* < 0.001) demonstrating that while cues were effective in influencing behavior, face and house discriminations were differentially modulated by cued expectations.

Several category-specific effects of expectation on face/house accuracy were replicated from previous work^14^, including a significant main effect of cue (*F*(1.32, 23.79) = 4.63, *p* < 0.03), but not task stimulus (*F*(1, 18) = 2.291, *p* = 0.15), on choice accuracy (see Fig. 2a). Consistent with our previous behavioral findings, face expectations strongly influenced house accuracy, whereas house expectations had relatively little impact on face accuracy. However, in contrast with our past study^14^, we found a significant accuracy advantage of house over face stimuli in the neutral (50%) cue condition (*t*(18) = 2.88, *p* = 0.01), suggesting a response bias for the house category. The main effects of cue (*F*(1.99, 35.93) = 14.92, *p* < 0.0001) and stimulus (*F*(1, 18) = 115.41, *p* < 0.0001) on RT were also consistent with our previous findings, such that RT for each category was influenced by cued expectations, and correct face responses were significantly faster than correct house responses, regardless of cue (Fig. 2b).

### Comparison of Model Fits

Next, we tested whether the observed behavioral biases were best supported by changes in the starting point (prior bias model: PBM), or drift-rate (dynamic bias model: DBM), or by changes in both parameters (multistage model: MSM). Consistent with our previous findings^14^, we found that, compared to fits of the PBM (DIC = 26723.87, Deviance = 26,593.57, *pD* = 130.30) and DBM (DIC = 26,708.33; Deviance = 26,666.99, *pD* = 41.33), the observed behavioral effects were best explained by the MSM despite the larger complexity penalty (DIC = 26,654.92, Deviance = 26,448.90, *pD* = 206.02). To ensure that the MSM provided reasonable qualitative predictions of the observed effects of cue validity on the mean accuracy and RT estimates shown in Fig. 2a,b, we overlaid the predicted values of each measure for 100 simulated datasets, each generated by concatenating simulated datasets for each subject based on their estimated parameters. Indeed, model predictions (transparent lines in Fig. 2a,b) fell within the 95% confidence intervals of all observed means for both accuracy and RT, providing additional support for combined expectation bias in the starting-point and drift-rate parameters. Finally, as a more direct assessment of goodness-of-fit, we compared the shape of observed and model-predicted RT distributions on correct and error trials for each category and across cue-type (Fig. 2c). Again, the high degree of similarity between observed and predicted RT distributions across all conditions bolsters our previous findings^14^ and current conclusions favoring a multi-stage mechanism by which prior information biases both the starting point (e.g., pre-sensory) and drift-rate (e.g., post-sensory) of visual category discriminations.

Based on our previous findings^14^, we next tested the following hypotheses: (1) Probabilistic cues will shift the starting point closer to the expected category boundary, “upwards” for face expectations and “downwards” for house expectations, relative to the neutral condition. (2) Starting-point estimates will reflect a general bias in favor of the face category, suggesting a stronger weighting of the prior than of sensory information for face decisions. (3) Face drift-rates will be higher following valid compared to invalid and neutral cues; whereas (4) house drift-rates will be lower on invalid compared to valid and neutral cues.

A comparison of the posterior distributions for the starting-point estimates in each of the cue conditions supported hypotheses 1 and 2 (Fig. 2e). Relative to the starting-point in the neutral condition (*z*_*n*_ = 0.66), house cues caused a significant downwards bias in the starting point (P(*z*_*h*_ <* z*_*n*_) = 0.99), whereas the upwards bias caused by face cues was only marginally significant (P(*z*_*f*_ > *z*_*n*_) = 0.91). However, in line with hypothesis 2, all starting-point estimates fell above the midpoint between boundaries (*z*_*all*_ > ½*a*), implying that subjects were generally biased towards expecting faces, with the strength of this bias modulated by cue type.

To ease interpretation and comparison of cue effects on drift-rates between categories, all tests were performed on the absolute values of the drift-rate (e.g., higher numbers always reflect faster drift-rates). In contrast to our previous findings (see hypothesis 3 above), we found that the drift-rate for face stimuli (Fig. 2f) was not significantly modulated by the preceding cue (P(*v*_*fF*_ > *v*_*nF*_) = 0.48; P(*v*_*fF*_ > *v*_hF_) = 0.77). However, consistent with hypothesis 4, the drift-rate for house stimuli (Fig. 2g) was significantly slowed following face cues, compared to trials with neutral cues (P(*v*_*fH*_ <* v*_*nH*_) = 0.98) and valid house cues (P(*v*_*fH*_ < v_*hH*_) = 0.99. Furthermore, consistent with our previous findings, valid house expectations did not improve house drift-rates compared to the neutral condition (P(*v*_*hH*_ < *v*_*nH*_) = 0.58). Overall, these findings support our hypotheses and closely match the observed starting-point and drift-rate dependencies on prior face and house expectations reported in an earlier study that used the same face/house discrimination task^14^.

### Imaging Results

**Figure 3:**
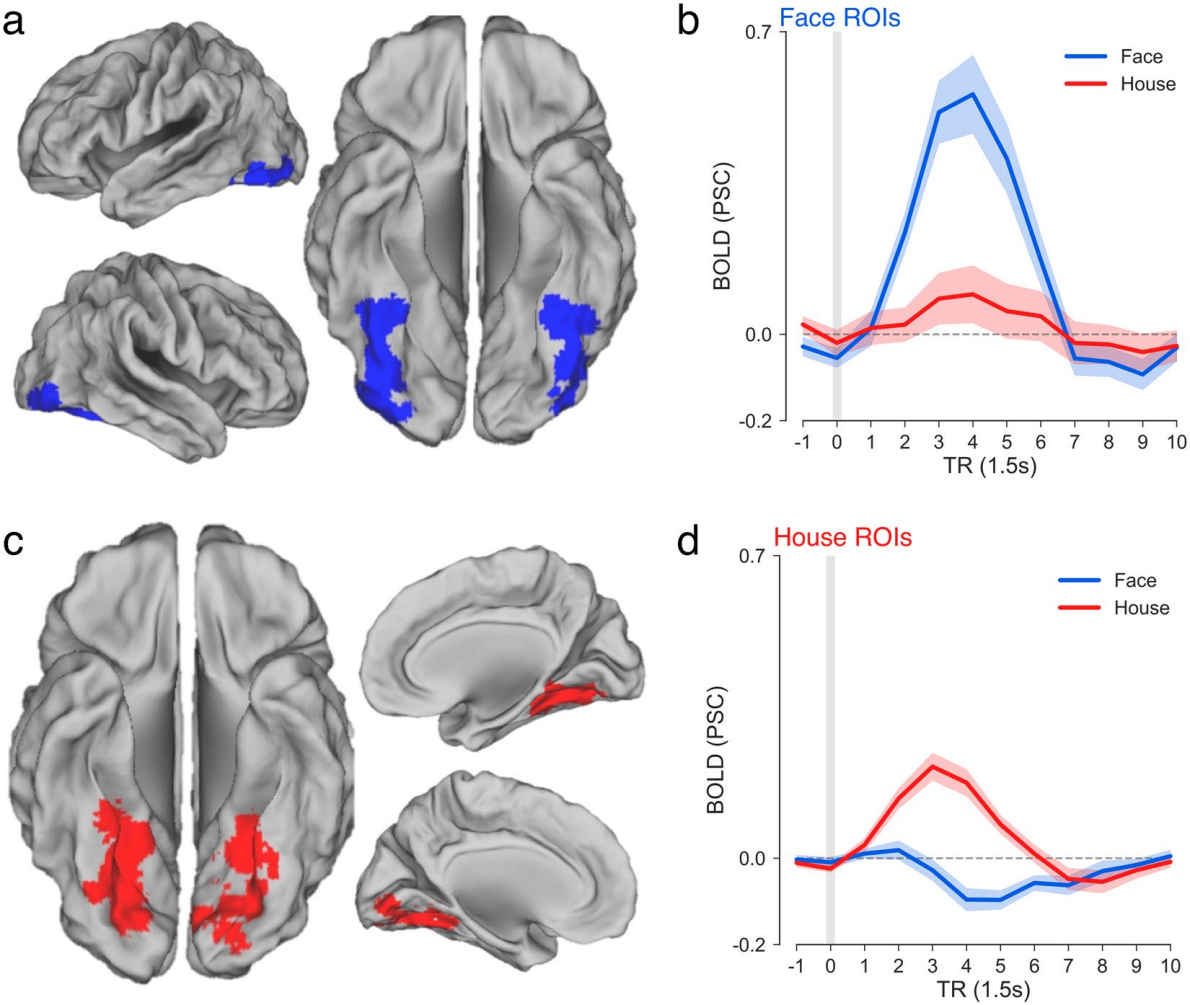
Category-selective regions and activity in ITC. (**a**) Face-selective ROIs, shown as a single contiguous cluster including all individual subject-localized face ROIs (N = 19). The average face-selective ROI across subjects was 349.73 voxels (SEM = 59.02). (**b**) Average timecourse of stimulus-evoked activity in face ROIs on correct face (blue) and house (red) trials. (**c**) House-selective ROIs (M = 364.32 voxels, SEM = 68.29) and (**d**) stimulus activation timecourses in house ROIs with same plotting conventions as in (**b**). Surface mapping was performed with Caret^45^ and the SuMS database^46^. Shaded area reflects +/−1 s.e.m.

Because of their involvement in evidence accumulation during face/house discrimination^1^, analysis of fMRI data focused on face- and house-selective regions of interest (ROI) in ITC, localized individually for each subject (see Methods). The task used two types of trial to permit a clean separation of cue-evoked and task-evoked fMRI data^15^. Full trials included both the cue and stimulus, and catch trials included only the cue. We extracted cue- and stimulus-evoked timecourses from face-preferential (Fig. 3a) and house-preferential ROIs (Fig. 3c) for each subject based on two different GLMs. The “separated-events” GLM separated cue and stimulus events using a cue-locked regressor for each cue-type (including cues from catch and full trials) and stimulus-locked regressors for each stimulus-type. The “combined-events” GLM included a cue-locked regressor for each cue-type on catch trials only, and a cue-locked regressor for each cue-stimulus combination on full trials. The purpose of the combined-events GLM was primarily to validate the effectiveness of the separated-events GLM. We also ran a second version of the separated-events GLM, in which stimulus events were coded by Fast (>median) and Slow (<median) RTs, to attempt to replicate the finding that the rising slope of the BOLD signal in ITC was steeper on Fast than Slow RT trials, a pattern that is consistent with an accumulation-to-boundary account (see Supplementary Materials)^1,16^.

Collapsing across the three cue regressors in the separated-events GLM, we extracted the stimulus-locked timecourse for Face and House stimuli to visualize the categorical preference for each set of regions. The face- and house-selective regions from all subjects are shown in Fig. 3a,c. As expected because of the contrast defining the ROIs, activation in face-selective regions showed a stronger positive deflection on Face than House trials (*F*(1,18) = 43.52, *p* < 0.0001; Fig. 3b). In contrast, the main effect of stimulus in house-selective regions was driven by a positive deflection from baseline on House trials and a negative deflection on Face trials (*F*(1,18) = 39.45, *p* < 0.0001; Fig. 3d).

### Effects of expectations on cue- and stimulus-evoked activity

**Figure 4:**
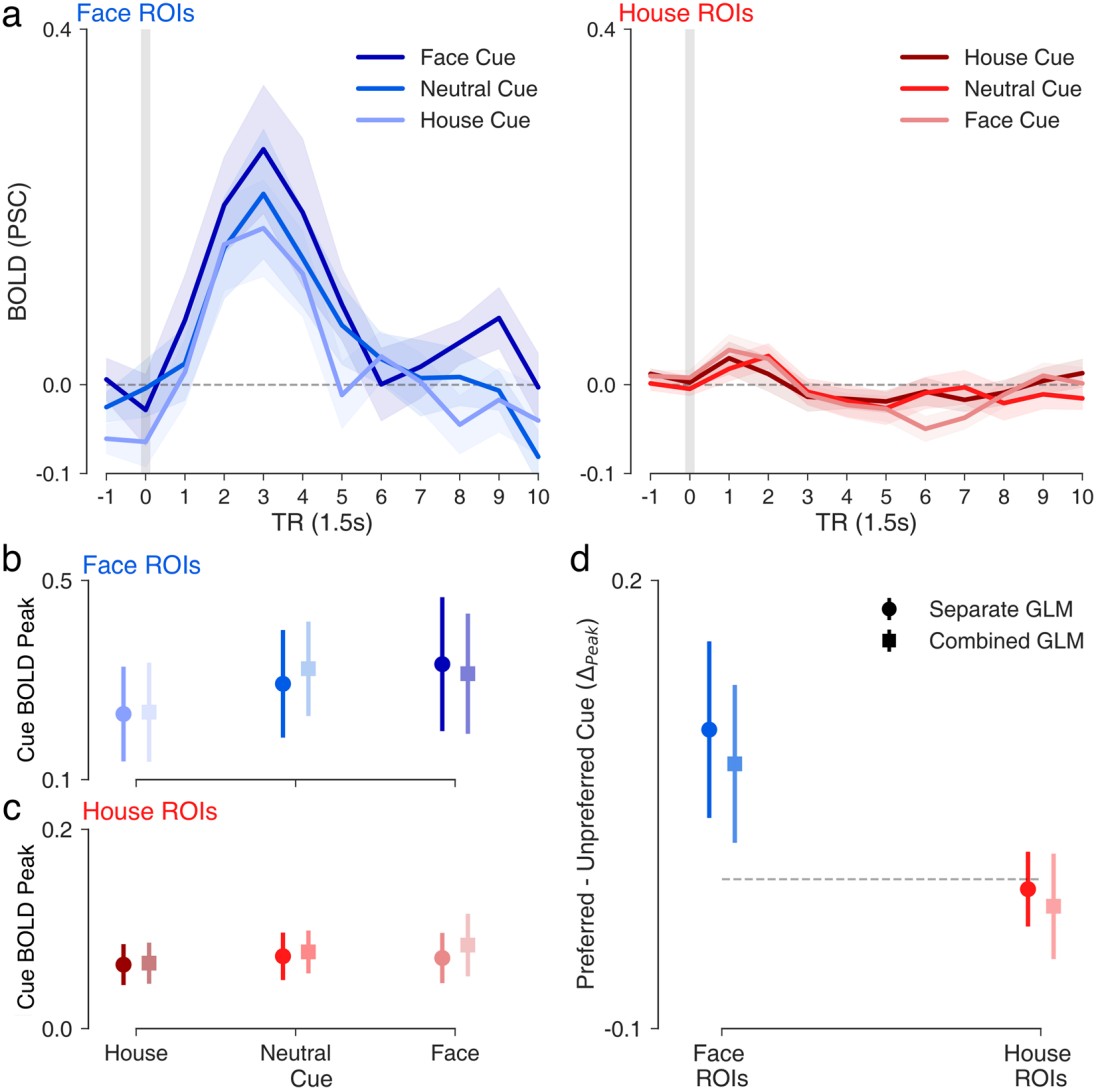
Effects of expectations on cue-phase activity. (**a**) Timecourse of activity (12 × 1.5 s) in face (blue, left) and house (red, right) ROIs time-locked to 1 TR before cue onset, estimated from from catch trials and full trials in the separate events GLM. Peak magnitude of activity plotted for each cue type in (**b**) face and (**c**) house ROIs based on estimates from the separate events GLM (circle markers; darker colors) and combined events GLM (square markers; lighter colors). (**d**) Peak difference in cue-evoked activity for region-preferred cues versus non-preferred cues in face (blue) and house (red) ROIs. Shaded area around mean timecourses reflect +/−1 s.e.m. Error bars in point plots reflect 95% c.i. around the mean.

Based on our previous study of prior expectations on face vs. house discrimination^14^, and on the parameter estimates in the MSM, we had specific a priori hypotheses about the cue-evoked activity in face- and house-selective regions of ITC. We predicted that face-selective regions would show stronger activity in the cue phase as the prior probability of a face stimulus increased, whereas house-selective regions would not reflect expectation-related activity in the cue phase. To test these predictions, we examined cue-evoked activity in face- and house-selective regions (Fig. 4a), as well as the peak magnitudes of cue-evoked activity in face and house-selective regions from the separated- and combined-events GLMs (Fig. 4b,c). Note that, due to the nature of the catch-trial design, there were far fewer catch-cue trials and thus fewer events in the combined GLM cue regressor than that of the separate GLM. In spite of this, the cue-evoked peaks estimated for the two model types were highly similar in face- and house-selective regions (Fig. 4b,c) indicating that the catch-trial design added sufficient variability between cue and stimulus events to isolate activation specific to each phase of the trial. The subject-wise peak magnitude for each cue type on catch and full trials was also highly correlated in face-selective (all *r* > 0.88, *p* < 0.0001) and house-selective regions (all *r* > 0.73, *p* < 0.0003), providing confirmatory evidence that the catch-trial design successfully isolated cue- from stimulus-evoked activity. Thus, all remaining analyses were conducted on data derived from the separated-events GLMs.

**Figure 5:**
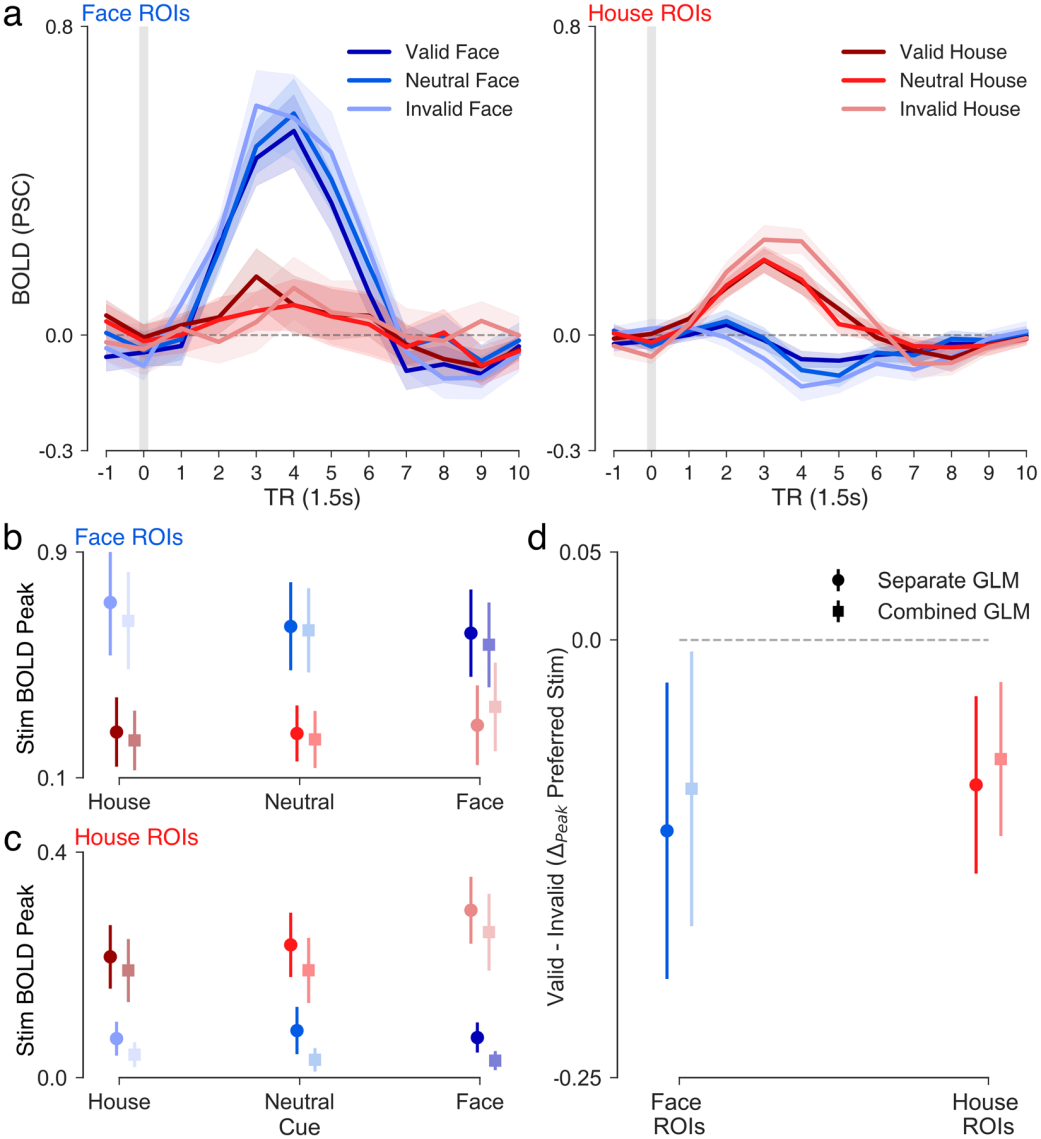
Effects of expectations on stimulus-phase activity. (**a**) Timecourse of activity (12 × 1.5 s) in face (blue, left) and house (red, right) ROIs time-locked to 1 TR before stimulus onset estimated from full trials in the separate events GLM. (**b**) Peak face-evoked activity in face-selective ROIs across cue types based on the separate events GLM (circle markers; darker shades) and combined events GLM (square markers; lighter shades). (**c**) Peak house-evoked activity in house-selective regions with same plotting conventions as in b. (**d**) Peak difference in stimulus-evoked activity following valid versus invalid expectations in face (e.g., left; blue error bars) and house (e.g., right; red error bars) ROIs. Negative values imply suppression of stimulus-evoked activity when cued expectations are valid. Shaded area around mean timecourses reflect +/−1 s.e.m. Error bars in point plots reflect 95% c.i. around the mean.

Cue-dependent time series data from face- and house-selective regions are shown in Fig. 4a. Only correct trials are included. Valid trials are those in which the cue and stimulus matched, invalid trials are non-matches (e.g., in an Invalid Face trial, an 80H cue is followed by Face stimulus). Consistent with our model-based predictions, the peak magnitude of cue-evoked activity in face-selective ITC (Fig. 4b) showed a pattern consistent with the mean starting-point estimates across cue types (e.g., house cue < neutral cue < face cue; see Fig. 2e). Face cues evoked a significantly greater peak magnitude of activation than house cues (all trials, Fig. 5c: *t*(18) = 3.26, *p* = 0.004; catch-trials, Fig. 5e: *t*(18) = 2.88, *p* = 0.01), but not neutral cues (all trials, Fig. 5b; *t*(18) = 0.64, *p* > 0.05; catch-trials, Fig. 5c: *t*(18) = −0.26, *p* > 0.05). Moreover, in line with the general face bias observed starting-point estimates, cue-evoked peak activation magnitudes for all cue types were significantly greater than baseline in face-selective regions (all p < 0.0001). House-selective regions in ITC showed a small positive deflection during the cue phase that was equal in magnitude for house- and face-predicting cues (Fig. 4d).

The pattern of stimulus-evoked activations from the separated events GLM in face- and house-selective regions (Fig. 5a) was consistent with model-based predictions, with unexpected stimuli evoking stronger responses than expected stimuli (Fig. 5d) in the corresponding area of ITC. To test the interaction between cued expectations and observed stimuli, we performed a 2 (Stimulus: Face, House) × 3 (Cue: House, Neutral, Face) repeated measures ANOVA on peak stimulus-evoked magnitudes extracted from face and house ROIs (separated-events GLM). Indeed, a significant interaction was detected in both face (*F*(2,36) = 3.46, *p* = 0.04) and house-selective (*F*(2,36) = 7.19, *p* = 0.002) regions, indicating that stimulus-evoked responses in both sub-regions of ITC were modulated by the preceding cue. The observed interaction in face ROIs is consistent with a predictive coding (PC) account in which the sensory response to a face stimulus is suppressed by valid face expectations (Fig. 5d). Interestingly, house-selective regions reflected expectation-related modulations in activity for both stimulus types, with unexpected faces evoking a stronger negative deflection than expected faces (*t*(18) = 4.74, *p* = 0.0002) and unexpected houses evoking a stronger positive deflection than expected houses (Fig. 5d; *t*(18) = 3.15, *p* = 0.006).

## Discussion

The primary aim of this study was to investigate the pre- and post-stimulus manifestation of probabilistic expectations in category selective areas of ITC, using a cuing paradigm to disambiguate signals related to top-down predictions and bottom-up prediction-errors. To this end, we leveraged computational modeling to quantify the effects of probabilistic cues on the baseline and rate of evidence accumulation in a face-house discrimination task and compared these estimates with cue- and stimulus-evoked neural activity measured by fMRI. In line with our main hypothesis, probabilistic cues led to a pre-stimulus *prediction* signal in category-selective ITC that increased with the strength of expectations, followed by a stimulus-evoked *prediction-error* signal that diminished with stronger expectations. The results are consistent with the role of bias in an accumulation-to-boundary account of information processing in which expectations modulate the magnitude of activation prior to the appearance of the stimulus, moving it closer to or further from a hypothetical decision boundary^17^.

Over the past decade, perceptual decision-making research has made clear the importance of expectations. Informative cues given prior to a perceptual judgment give rise to preparatory neural activity in task-relevant sensory^13,15^, motor^18–20^, and associative^21,22^ cortices. Interestingly, a number of neuroimaging studies have found that prior expectations often have the opposite effect on stimulus-evoked activity, suppressing the response to predictable stimuli and amplifying the response to surprising ones. PC theories posit that the amplified response to surprising stimulus reflects the *error* between predicted and observed sensory features. Unfortunately, because of the low temporal resolution of fMRI it has proven difficult to dissociate activity related to the *prediction error* from activity related to the *prediction* itself. As a result, most evidence supporting PC comes from sensory-evoked patterns in stimulus specific areas of cortex whereas relatively little is known about how expectations affect these areas prior to stimulus onset. Moreover, in the few imaging studies that have identified prediction-related signals in sensory cortex^13,15^, the subsequent stimulus-evoked response has been found to be amplified, not suppressed, by valid expectations.

For instance, Puri *et al*.^13^ measured brain activity using fMRI during a cued face/house discrimination task which differed from the current study in that predictive cues were followed by a constant six second delay before stimulus onset. Similar to the findings presented here, these authors observed a cue-evoked baseline shift in face-selective ITC, greater when a face was explicitly expected. However, in contrast with the results of the current study^13^, they found that activity aligned to the onset of the stimulus was greater in face- and house-selective regions when expectations were validated by the region-preferred stimulus. Thus, prior expectations amplified rather than suppressed stimulus-evoked activity in face and house-selective areas of ITC. One explanation for this discrepancy is that, due to the regular (i.e., un-jittered) interval between cue and stimulus events in Puri *et al*.^13^, the amplified activity following expected stimuli could reflect residual activity from the cue-phase. The catch-trial design employed in the current study allowed us avoid this potential confound, providing a reliable dissociation of cue- and stimulus-evoked activity. Consistent with a PC interpretation, we observed that category-preferential regions of ITC displayed a greater response to surprising than to expected stimuli. In face-selective ITC, this apparent prediction error signal was preceded by an increase in activity proportional to the cued probability of a face. Together, these findings suggest that, at least in some cases, the amplified response to surprising sensory input reflects a greater discrepancy between the expected and observed features of a stimulus.

We should note that the expectation-enhancing effects observed by Puri *et al.*^13^ are not necessarily artefactual, and, in fact, there is good reason to believe that stimulus processing involves concurrent prediction and surprise signals throughout the visual hierarchy^11,23^. According to this view, valid expectations would correspond to an increase in the signal of prediction units but a decrease in the signal of surprise units. Based on a computational model aimed at decomposing the relative contribution of these two mechanisms, Egner and colleagues^4^ estimated roughly a 2:1 ratio of surprise to prediction units within a given region (see also Supplementary Fig. 3). Thus, even though expectation suppression is thought to be more prevalent, there are a number of methodological differences between studies that may account for the observation of expectation enhancement. As mentioned above, one working hypothesis^23,24^ is that, in the context of valid expectations, enhanced stimulus-evoked responses reflect carryover of the top-down prediction signal, driving an increase in the BOLD signal that extends into the stimulus phase. Still, expectation enhancement has also been observed in fMRI studies, such as this one, that use a catch trial design to isolate cue- and stimulus-evoked signals^15^. One possibility is that the apparent amplifying effect of expectations on sensory signals in these studies is the result of attentional gain on task-relevant prediction errors^25–27^, offsetting the effect of error suppression.

The findings of the current study are largely in line with the assumptions of PC theories, and, perhaps more importantly, they also point to certain benefits of accumulation-to-bound models. For instance, PC theories are generally agnostic about processing duration and do not offer a straightforward account for the RT-dependent rise of activity in ITC observed in this (see Supplementary Fig. 1) and a previous study^1^, in which faster decisions correspond to a steeper rising slope and earlier onset of decay compared to slower decisions. One possibility is that ITC is only active as long as the information it is propagating forward is goal-relevant – that is, until sufficient evidence has been accumulated to commit to a choice^1,16^ – implicating ITC in the accumulation of sensory *evidence* during goal-directed behavior, as opposed to simply responding to bottom-up sensory inputs. To this point, it is important to note that all trials lasted 6 sec, and stimuli remained on screen throughout the trial regardless of when the response was made. Despite all trials involving the full 6 sec duration of sensory stimulation, face and house-selective regions exhibited both a steeper slope and an earlier decay in activation on trials with fast compared to slow RTs (see Supplementary Fig. 1c). Additionally, behaviorally-derived biases in the starting-point and drift-rate parameters provided category-idiosyncratic predictions of mean cue- and stimulus-evoked activation in face- and house-selective regions, and were correlated with cue and stimulus-evoked magnitudes across individual subjects (see Supplementary Fig. 2). At the group level, cues manifested in varying degrees of face bias in the starting-point (e.g., *z*_*f*_ > *z*_*n*_ > *z*_*h*_; *z*_*all*_ closer to the face boundary) and pre-stimulus BOLD activity in face -selective regions. In contrast, expectations selectively biased the drift-rate of house decisions and led to a more pronounced modulation of stimulus-evoked activity in house-selective, compared to face-selective, regions. Other findings, however, do not appear to be well explained by either theoretical framework. For instance, stimulus-evoked activity in house-encoding regions displayed the same interaction between expected and observed input as face-selective regions, despite the apparent lack of an explicit “house” expectation signal in the cue phase (see Fig. 4a). One possibility is that prediction-error signals are not necessarily dependent on the existence of an explicit prediction template in the same region, and may arise from comparison between local sensory afferents and another source of top-down or lateral feedback.

In line with previous findings^14,28^, several observations in the current study suggest that these category-specific forms of bias may have resulted from strategic differences in the way subjects treated face and house stimuli – recasting the face-house discrimination as a match (face)/mismatch (house) judgment, or face/not-face. First, we found no evidence that subjects formed explicit house predictions in either in cue-evoked activity of house-selective areas (Fig. 4a) or in starting-point estimates of the DDM (Fig. 2d). Secondly, in line with our previous findings, house decisions were associated with stronger overall drift-rates than face decisions (P(*v*_*H*_ > *v*_*F*_) ≥ 0.995 for all cues). In a face/not-face scenario, the drift-rate on correct “mismatch” (e.g., house) trials is driven by two sources of evidence (e.g., house evidence + noise), compared to the single source of evidence available on correct “match” trials (e.g., face evidence). Finally, the negative signal deflection on correct face trials in house-selective regions (Fig. 5a) was proportional to, and slightly lagged behind, the activation in face selective regions. This pattern is consistent with a face-centric decision strategy in which house evidence (as indexed by the moment-to-moment level of activation in house-preferential regions) is reactively suppressed when surprising face inputs are detected, increasing in strength as the magnitude of the face prediction error increases. Finally, we estimated the relative contribution of prediction and error units to BOLD activation in both category-selective ITC regions by fitting the predictive coding model proposed by Egner *et al*.^4^ to event-related peaks extracted from the combined-events GLM (e.g., cue and stimulus phases coded as single cue-locked events). Model fits were largely consistent with the patterns reported in Egner *et al*.^4^ for both face- and house-selective ROIs, both regions displaying a stronger contribution of error, compared to prediction, units to the aggregate cue-stimulus response (see Supplementary Fig. 3). Consistent with our above interpretation that top-down expectations were stronger for the face category, face-selective regions displayed a stronger contribution from prediction units than did house-selective regions (see Supplementary Materials for additional details).

In conclusion, these findings provide insight into the probabilistic nature of neural prediction signals and their influence on the processing of subsequent sensory inputs in category selective regions of ITC, and how this interaction drives behavioral choice. It has become increasingly clear that decision-making computations are widely distributed throughout the brain^29,30^ (see Supplementary Fig. 4 and Supplementary Table 1 for a summary of whole brain cue and stimulus effects), involving complex circuit- and network-level dynamics that are abstracted, but ultimately unaccounted for, by accumulator models like the DDM^21,31^. Still the DDM provides a parsimonious, mechanistic account of decision-making that is capable of describing both RT and accuracy^32^ with reliable, identifiable^33^ parameter dependencies. On the other hand, sufficiently detailed accounts of PC^2,34^ offer a more biologically plausible set of predictions for guiding experimental investigation of the neural mechanisms underlying parameter changes in the diffusion model. However, increasing biological plausibility means sacrificing behavioral tractability; thus, it is of interest to theoreticians at the cognitive (algorithmic) and neural (implementation) levels to identify points of compatibility between these two promising frameworks.

## Materials and Methods

### Subjects

Twenty-four healthy (n = 24), right-handed, native English speakers with normal or corrected-to-normal vision participated in a 1.5-hour behavioral and functional magnetic resonance imaging (fMRI) session. Five subjects were excluded for incomplete scan data (N = 3) or insufficient behavioral data in all conditions for reliable analysis (N = 2). The remaining 19 subjects (9 female) ranged in age from 21 to 29 years (mean 24.7). All methods and procedures were approved by the Institutional Review Board (IRB) at the University of Pittsburgh and were carried out in accordance with the approved protocol. Informed consent was obtained from all subjects following the guidelines of the IRB. Subjects were compensated at a rate of $10 per hour for their participation. All behavioral and imaging data will be made available upon request (see contact information for the corresponding author).

### Stimuli

The stimulus set consisted of 42 neutral-expression face images (MacBrain Face Stimulus Set, provided MacArthur Foundation Research Network on Early Experience and Brain Development, Boston, MA) and 43 house images (photographs of houses taken in the local Pittsburgh area). Face and House images were cropped to remove non-relevant features, all with the same dimensions (512 × 512 pixels). Face and house stimuli were then normalized, noise-degraded, and converted into movies 1,14. Each original image was degraded by filtering the signal in the original stimulus (normalized brightness, luminance, and contrast) with a Gaussian white noise kernel using parameters that yielded a new version of the original image with a desired signal to noise ratio (SNR). This procedure produced a single still frame of the original image that was embedded in randomly distributed noise. Repeating this procedure 90 times for image X, at a given noise level, and concatenating the set of images X_1_–X_90_, produced a dynamic display of noise over the face or house stimulus. All 90 still frames, each containing a random spatial distribution of noise of the same SNR, were presented at 15 frames per second to produce a movie running 6 seconds in length. The image processing procedure was repeated for all individual face and house stimuli.

It is important to note that, while the spatial pattern of noise over the image changes from frame to frame, the SNR of the image is kept constant for the full duration of the trial (6 s). In contrast with similar paradigms in which the stimulus is gradually revealed over the course of each trial, the current approach of holding the SNR constant allows neural signatures of evidence accumulation to be investigated independently from basic sensory processing. Based on previous work^1,14^ we fixed the noise-level of face and house images at 67%. One approach is to psychophysically matched noise levels across the two types of stimuli in order to control for category-specific effects. However, one original aim of the current study was to examine differences in the effects of expectation for categories with more (i.e. face) and less (i.e. house) predictable features^14^. Thus, face and house stimuli were degraded to include the same level of noise in order to preserve category specific effects.

### Task

In the task, subjects were presented with noise-degraded movie stimuli for 6 sec and made a face versus house discrimination decision at any time during trial. Each test trial, which we will refer to as the “task phase”, was preceded by a cue (in the “cue phase”) informing the participant of the probability (i.e., the prior) that the upcoming stimulus would be a face or a house (Fig. 1). The cue phase consisted of a screen-centered movie of 99% noise surrounded by a red border. Cues appeared as a number-letter combination in the center of the movie frame. Before scanning, subjects were trained on how to interpret the cues. The complete list of cues included an 80% prior for face (80F) and house (80H) and a 50% neutral cue (50N) indicating an equal probability for each category. After the three second cue phase, the border turned green to indicate trial onset and an image was present within the dynamic field of noise. Subjects were given six seconds to make a response indicating their choice with a button press using the left or right index fingers. Jittered periods between trials (see *Design and Timing*) consisted of a 99% noise degraded movie surrounded by a grey border, during which the subject was instructed to rest until presentation of the subsequent cue. The laterality of stimulus-response mapping was counterbalanced across subjects. Cues were valid predictors of the probability of seeing each category on the upcoming task phase (see *Design and Timing*). Stimulus presentation and behavioral data collection were programmed using the PsychoPy software package^35^. Prior to beginning the main experiment, subjects were given a chance to practice the task for a maximum of 15 minutes, less if they were comfortable with the instructions.

### Design and Timing

In the experiment, subjects completed a total of 600 trials split across five runs of 120 trials per run in a rapid event-related design. In order to reliably separate activation related to cue and task events, a catch-trial design^15,36–38^ was used in which 20% of trials were cue-only (i.e. catch-trials). Thus, of the 200 trials for each cue type (80F, 50N, 80H), 40 were catch-trials (Fig. 1, bottom; three seconds total duration), in which the cue phase was immediately followed by a brief inter-trial interval, indicated by a gray border and 99% noise stimulus. Subjects were informed of this condition prior to the experiment and told that this additional contingency did not impact the reliability at which the cues predicted each stimulus. The remaining trials were full trials, consisting of both the cue and task phases (Fig. 1, top; nine seconds total duration). On full trials, the cue veridically indicated the probability of seeing a face or house stimulus. For example, on full trials in the 80F condition, the cue was followed by a face stimulus on 128 trials (i.e., valid trials) and by a house stimulus on 32 trials (e.g., invalid trials). Full trials in the 50N condition included 80 face and 80 house stimuli.

Using a standard rapid event-related design^39^, trial onset times were jittered such that duration of the rest periods were distributed exponentially, ranging between 0 and 15 second durations (mean = 1.92 seconds) at multiples of the repetition time (TR; see Image Acquisition). The specific order and temporal distribution of rest periods was generated using optseq2, a tool that optimizes the ordered sequence of conditions for minimal covariance between regressors^39^. The catch trial approach eliminates the need to jitter the cue and stimulus phase onsets on full trials, thus stimulus onset time in the task phase was fixed at the end of the cue phase.

### Image Acquisition

Functional and anatomical images were acquired on a Siemens Allegra 3-Tesla scanner. High-resolution anatomical images were acquired using a T1-weighted MP-RAGE sequence (repetition time [TR] = 1.54 s, echo time [TE] = 3.04 ms, flip angle [FA] = 8 degrees, inversion time [TI] = 800 ms). T2-weighted anatomy images were obtained with a spin-echo sequence (TR = 6.0 s, TE = 73 ms, FA = 150 degrees, 38 slices). Functional images sensitive to the BOLD contrast were acquired with a whole-brain echo-planar T2*-weighted series (TR = 1.5 s, TE = 25 ms, FA = 60 degrees, 3.125 × 3.125 mm in-plane resolution, 3.5 mm slice thickness, 29 slices). The first four images of a run were discarded to allow net magnetization state and RF signal to approach steady state. Subjects were provided with earplugs to minimize scanner noise. T1- and T2-weighted images were used primarily to convert functional data to atlas space.

### Behavioral Data Analysis

Fast RT outliers were removed from data for each subject using the exponentially weighted moving average (EWMA) analysis packaged in D-MAT, an open source MATLAB toolbox for fitting diffusion models^40^. In total, 1.94% of trials were eliminated based on subject-specific EWMA estimates. Mean RT (correct trials only) and accuracy measures were analyzed separately using a 2 (stimulus: face, house) × 3 (cue: 80H, 50N, 80F) repeated-measures ANOVA model. Finally, a one-way repeated measures ANOVA was run within face and house categories separately to test the simple effect of probabilistic cues on RT. All analyses revealed that the data significantly violated the sphericity assumption. Thus, Greenhouse-Geisser corrected results are reported for both measures. All pairwise comparisons reported are corrected for multiple comparisons using a Sidak HDR correction.

### Drift-Diffusion Model (DDM)

The Drift Diffusion Model (DDM) assumes that binary decisions are made by accumulating stochastic evidence in a decision variable (DV) between two decision boundaries, each representing the evidence criterion for choosing one of two categorical outcomes. As evidence builds for one category over the other, the DV drifts towards favored boundary until reaching criterion, executing the corresponding choice (e.g., accuracy) and marking the time of decision (e.g., RT) of a given trial. Repeating this process many times yields a response time distribution for each category as well as the proportion of correct and incorrect decisions, accounting for commonly observed speed-accuracy tradeoffs that are unaccounted for by standard behavioral analyses.

Models were fit to the data using HDDM^41^, a python package for hierarchical Bayesian optimization of DDM parameters. Hierarchical Bayesian parameter estimation enables simultaneous, nested estimation of group- and individual subject-level parameters where each parameter is represented as a distribution reflecting the uncertainty over a range of possible values. In this form of hierarchical model, group-level uncertainty associated with each parameter is derived from variability between subject-level estimates, and in turn, forms the prior distribution from subject-level posterior distributions are sampled. Given the likelihood function *L*(parameters|data), Bayes rule is applied to update the prior distribution of each parameter to reflect the new estimate of uncertainty given the observed trial-wise RT and accuracy data. Thus, for each parameter, model fits return a posterior distribution which can be directly used to evaluate the statistical reliability of condition effects on a given parameter. For instance, to test the hypothesis that face cues significantly biased the starting-point relative to the neutral condition, assuming a criterion of 5% probability that the observed effect is due to chance, a significant difference would be reported if the overlap of the posteriors was equal to or less than 5%.

All models were constructed such that a face (house) response resulted when a positive (negative) drift-rate caused the DV to terminate at the top (bottom) decision boundary. Defining decision boundaries with respect to category rather than accuracy, which is typically done for binary decision tasks, is important for meaningful interpretation of an upwards (i.e., face) or downwards (i.e., house) expectation bias in the starting-point parameter. All models included group- and subject-level estimates for boundary height (*a*), face drift-rate (*v*_*F*_), house drift-rate (*v*_*H*_), starting-point (*z*), and non-decision time (*tr*). Parameter estimates of inter-trial variability in the drift-rate (*s*_*v*_), starting-point (*s*_*z*_), and non-decision time (*s*_*t*_) were included at the group-level only, as reliable estimation of these parameters requires more observed trials than available in individual subject datasets. We fit three versions of the drift-diffusion model to choice and RT data in which probabilistic cues were assumed to bias the face/house discrimination by modulating either the starting-point, the drift-rate, or both. We refer to the model allowing only the starting-point to vary across conditions as the prior bias model (PBM) and the model allowing only the drift-rate to vary across cues as the dynamic bias model (DPM), as a change in the starting-point implies a pre-sensory source of bias whereas a change in the drift-rate biases the accumulation of sensory input dynamically. Finally, we refer to the model in which both parameters are free to vary across conditions as the multi-stage bias model (MSM).

To identify parameters dependencies on cue and stimulus conditions, 80F, 50N, and 80H cues are denoted by lower case ‘f’, ‘n’, and ‘h’ subscripts and Face and House stimuli are denoted by uppercase ‘F’ and ‘H’ subscripts. Thus, in the PBM starting-point estimates for each cue are shown as *z*_*f*_, *z*_*n*_, and *z*_*h*_, and drift-rates for Face and House stimuli as *v*_*F*_ and *v*_*H*_. In the DBM, Face drift-rates in each cue condition are shown as *v*_*fF*_, *v*_*nF*_, and *v*_*hF*_, and House drift-rates as *v*_*fH*_, *v*_*nH*_, and *v*_*hH*_. In the MSM, starting-point (*z*_*f*_, *z*_*n*_, and *z*_*h*_), face drift-rate (*v*_*fF*_, *v*_*nF*_, and *v*_*hF*_), and house drift-rate (*v*_*fH*_, *v*_*nH*_, and *v*_*hH*_) parameters were free to vary across cue type. To account for differences in model complexity (proportional to the number of free parameters included compared to the others), model fits were compared using the Deviance Information Criterion (DIC), which reflects the sum of the Deviance (measure of discrepancy between data and model predictions) and a complexity penalty, pD (the effective number of parameters included in the model). Thus, models with a lower DIC are considered to provide a better fit to the data. Compared to more traditional measures of model fit (i.e. BIC, AIC) the DIC is better suited for assessing performance and complexity of hierarchical models, where both measures are nested at multiple levels. The accepted threshold for determining a significant difference between two models fit to the same data is a DIC of 10 or more^42^.

### Functional Imaging Preprocessing

Imaging data were preprocessed to address noise and image artifact. Preprocessing included within-TR slice timing correction using sinc interpolation, motion correction using rigid-body rotations and translations, within-run voxel intensity normalization to a mode of 1000 to facilitate inter-subject comparisons^43^, and computation of atlas space transformation matrices. Data were resampled to 2 mm isotropic voxels and transformed into stereotaxic atlas space during group analyses. All preprocessing and imaging analysis was carried out using proprietary software^37,38^ developed at Washington University in St. Louis.

### General Linear Models (GLMs)

Subject-level data were analyzed with voxelwise general linear modeling^44^ (GLM). Three GLMs were constructed in total. In the first GLM (the combined events GLM), full trials were coded by the combination of cue type and stimulus category and catch trials were coded by cue type only. This model isolated cue-evoked activity on the cue-only catch trials, thus eliminating possible stimulus-related contributions to the observed signal change during the expectation period, and estimated full trials as the combined effect of the cue and stimulus over the entire trial duration. The primary purpose of this model was to help validate data estimated using the separate events GLM. In the separate events GLM, the cue and stimulus phases of each full trial were treated as separate regressors. All occurrences of each cue type (e.g., 80F cues from catch and full trials) were aggregated into a single cue event type. Stimulus phase events were coded by a combination of stimulus category (face, house) and the preceding cue type (80H, 50N, 80F). This model takes full advantage of the catch trial design by isolating cue- and stimulus-evoked timecourses into separate response functions.

Models were fit to the data using a finite impulse response (FIR) method, which makes no assumption about the shape of the BOLD response. In the combined-events GLM, a series of 14 delta functions described event-related effects as a time series of the percent of BOLD signal change from baseline, time-locked to 1TR before cue onset for both catch and full trial types. In the separate events GLM, a series of 12 delta functions described event-related effects as a time series of the percent of BOLD signal change from baseline, time-locked to 1TR before cue onset for cue events and and 1TR before task onset for task events. We used software developed at Washington University in St. Louis for image processing and analysis^37,38^.

### Localization of face- and house-selective regions of ITC

Analyses pertaining to the main hypotheses focused on face- and house-selective regions of interest, satisfying the same criteria for object selectivity as in Tremel and Wheeler^1^. From the separated-events model, face- and house-preferential ROIs within the fusiform and parahippocampal gyri were defined for each subject by generating a voxelwise face-minus-house contrast map using two-tailed t-tests. This analysis generated subject-specific statistical maps showing all voxels with face- and house-preferential activations, collapsing across cue type and including only activation from the stimulus phase. These initial *t*-statistical maps were smoothed with a 4 mm full width at half maximum (FWHM) using a Gaussian kernel. Peak voxels of activity exceeding 95% confidence level (−1.96 > Z > 1.96) were identified, and an 8 mm spherical region of interest (ROI) was grown around each peak. Voxels that were absent from the initial statistical map (i.e., below the Z-statistic threshold) were dropped from the ROIs. Each subject contributed at least one face-preferential and one house-preferential region to subsequent group-level random-effects analyses examining cue and stimulus effects on the magnitude of regional activation.

## Electronic supplementary material

Supplementary Materials

## Data Availability

The datasets generated during and/or analyzed during the current study are available from the corresponding author upon request.

## References

[1] Tremel JJ, Wheeler ME (2015). Content-specific evidence accumulation in inferior temporal cortex during perceptual decision-making. Neuroimage.

[2] Friston K (2005). A theory of cortical responses. Philos. Trans. R. Soc. Lond. B. Biol. Sci..

[3] Summerfield C, de Lange FP (2014). Expectation in perceptual decision making: neural and computational mechanisms. Nat. Rev. Neurosci..

[4] Egner T, Monti JM, Summerfield C (2010). Expectation and surprise determine neural population responses in the ventral visual stream. J. Neurosci..

[5] Kok P, Jehee JFM, de Lange FP (2012). Less Is More: Expectation Sharpens Representations in the Primary Visual Cortex. Neuron.

[6] Chennu S (2013). Expectation and attention in hierarchical auditory prediction. J. Neurosci..

[7] Ulanovsky N, Las L, Nelken I (2003). Processing of low-probability sounds by cortical neurons. Nat Neurosci.

[8] Blakemore SJ, Wolpert DM, Frith CD (1998). Central cancellation of self-produced tickle sensation. Nat. Neurosci..

[9] Allen M (2016). Anterior insula coordinates hierarchical processing of tactile mismatch responses. Neuroimage.

[10] Zelano C, Mohanty A, Gottfried JA (2011). Olfactory predictive codes and stimulus templates in piriform cortex. Neuron.

[11] Bell, A. H., Summerfield, C., Morin, E. L., Malecek, N. J. & Ungerleider, L. G. Encoding of Stimulus Probability in Macaque Inferior Temporal Cortex. *Curr*. *Biol*. 1–11, 10.1016/j.cub.2016.07.007 (2016). 10.1016/j.cub.2016.07.007 PMC5021632 27524483

[12] Kok P, Failing MF, de Lange FP (2014). Prior Expectations Evoke Stimulus Templates in the Primary VisualCortex. J. Cogn. Neurosci..

[13] Puri AM, Wojciulik E, Ranganath C (2009). Category expectation modulates baseline and stimulus-evoked activity in human inferotemporal cortex. Brain Res..

[14] Dunovan KE, Tremel JJ, Wheeler ME (2014). Prior probability and feature predictability interactively bias perceptual decisions. Neuropsychologia.

[15] Shulman GL (1999). Areas involved in encoding and applying directional expectations to moving objects. J. Neurosci..

[16] Ploran EJ, Tremel JJ, Nelson SM, Wheeler ME (2011). High quality but limited quantity perceptual evidence produces neural accumulation in frontal and parietal cortex. Cereb. cortex.

[17] Hanes DP, Schall JD (1996). Neural control of voluntary movement initiation. Science.

[18] de Lange FP, Rahnev DA, Donner TH, Lau H (2013). Prestimulus oscillatory activity over motor cortex reflects perceptual expectations. J. Neurosci..

[19] Forstmann BU, Brown S, Dutilh G, Neumann J, Wagenmakers E-J (2010). The neural substrate of prior information in perceptual decision making: a model-based analysis. Front. Hum. Neurosci..

[20] Thura D, Cisek P (2016). Modulation of Premotor and Primary Motor Cortical Activity during Volitional Adjustments of Speed-Accuracy Trade-Offs. J. Neurosci..

[21] Filimon F, Philiastides MG, Nelson JD, Kloosterman NA, Heekeren HR (2013). How Embodied Is Perceptual Decision Making? Evidence for Separate Processing of Perceptual and Motor Decisions. J. Neurosci..

[22] Rahnev D, Lau H, de Lange FP (2011). Prior Expectation Modulates the Interaction between Sensory and Prefrontal Regions in the Human Brain. J. Neurosci..

[23] de Gardelle V, Waszczuk M, Egner T, Summerfield C (2013). Concurrent repetition enhancement and suppression responses in extrastriate visual cortex. Cereb. Cortex.

[24] de Gardelle V, Stokes M, Johnen VM, Wyart V, Summerfield C (2013). Overlapping multivoxel patterns for two levels of visual expectation. Front. Hum. Neurosci..

[25] Krauzlis RJ, Bollimunta A, Arcizet F, Wang L (2014). Attention as an effect not a cause. Trends Cogn. Sci..

[26] Jiang J, Summerfield C, Egner T (2013). Attention sharpens the distinction between expected and unexpected percepts in the visual brain. J. Neurosci..

[27] Summerfield C, Egner T (2009). Expectation (and attention) in visual cognition. Trends Cogn. Sci..

[28] Summerfield C, Egner T, Mangels J, Hirsch J (2006). Mistaking a House for a Face: Neural Correlates of Misperception in Healthy Humans. Cereb. Cortex.

[29] Churchland AK, Kiani R (2016). Three challenges for connecting model to mechanism in decision-making. Curr. Opin. Behav. Sci..

[30] Murakami M, Mainen ZF (2015). Preparing and selecting actions with neural populations: toward cortical circuit mechanisms. Curr. Opin. Neurobiol..

[31] Heitz RP, Schall JD (2012). Neural Mechanisms of Speed-Accuracy Tradeoff. Neuron.

[32] Ratcliff R, Smith PL, Brown SD, McKoon G (2016). Diffusion Decision Model: Current Issues and History. Trends Cogn. Sci..

[33] Ratcliff R, Tuerlinckx F (2002). Estimating parameters of the diffusion model: approaches to dealing with contaminant reaction times and parameter variability. Psychon. Bull. Rev..

[34] Rao RP, Ballard DH (1999). Predictive coding in the visual cortex: a functional interpretation of some extra-classical receptive-field effects. Nat. Neurosci..

[35] Peirce JW (2007). PsychoPy-Psychophysics software in Python. J. Neurosci. Methods.

[36] Wheeler ME (2006). Evidence for separate perceptual reactivation and search processes during remembering. Cereb. Cortex.

[37] Ollinger JM, Shulman GL, Corbetta M (2001). Separating processes within a trial in event-related functional MRI. I. The Method. Neuroimage.

[38] Ollinger JM, Corbetta M, Shulman GL (2001). Separating processes within a trial in event-related functional MRI (II. Analysis) 5080. Neuroimage.

[39] Dale AM (1999). Optimal experimental design for event-related fMRI. Hum. Brain Mapp..

[40] Vandekerckhove J, Tuerlinckx F (2007). Fitting the Ratcliff diffusion model to experimental data. Psychon. Bull. Rev..

[41] Wiecki TV, Sofer I, Frank MJ (2013). HDDM: Hierarchical Bayesian estimation of the Drift-Diffusion Model in Python. Front. Neuroinform..

[42] Spiegelhalter, D., Best, N. G. & Carlin, B. P. Bayesian deviance, the e ective number of parameters, and the comparison of arbitrarily complex models. (1998).

[43] Ojemann JG (1997). Anatomic localization and quantitative analysis of gradient refocused echo-planar fMRI susceptibility artifacts. Neuroimage.

[44] Friston K, Jezzard P, Turner R (1994). Analysis of functional MRI time-series. Hum. Brain Mapp..

[45] Caret (software). *Wikipedia*, *The Free Encyclopedia* (2015). Available at: https://en.wikipedia.org/w/index.php?title=Caret_(software)&oldid=670893255. (Accessed: 23rd August 2017).

[46] Van Essen, D. C., Harwell, J., Hanlon, D. & Dickson, J. Surface-Based Atlases and a Database of Cortical Structure and Function. *Databasing Brain From Data to Knowl*. 369–387 (2005).

