# Supplementary material for: Computational and neural signatures of pre and post-sensory expectation bias in inferior temporal cortex
Source: Sci Rep. 2018 Sep 5;8:13256. doi: 10.1038/s41598-018-31678-x (PMC6125426; doi:10.1038/s41598-018-31678-x)
Supplement: Supplementary file 1 — Supplementary Materials [file 41598_2018_31678_MOESM1_ESM.pdf]

Computational and neural signatures of pre and post-sensory expectation bias in inferior temporal cortex [SUPPLEMENTARY MATERIALS]

*Kyle Dunovan[1,2] and Mark E. Wheeler[3]

[1]Department of Psychology, [2]Center for the Neural Basis of Cognition, University of Pittsburgh, Pittsburgh, PA, USA. [3]School of Psychology, Georgia Institute of Technology, Atlanta, GA, USA.

RT-dependent rate of rise in face- and house-selective areas. In addition to the primary imaging analyses of expectation-related effects on ITC activation during the cue and stimulus phases, we fit another GLM in order to verify RT-dependent effects on the slope of the BOLD signal in face- and house-selective regions from a previous study[1]. The GLM included a regressor for cue type, time-locked to 1 TR before cue-onset, and a regressor for response speed, time-locked to 1 TR before stimulus onset (with each trial coded as either "fast" or "slow" based on by a median split of the RTs for each subject and stimulus category). The resulting timecourse of coefficients was then used to calculate the rising slope of activation on fast and slow trials - estimated by first interpolating 1000 time points between each TR to determine the time of activation onset and the time of activation peak. The time of activation onset was defined as the time at which 15% of the peak magnitude was reached. The slope of the interpolated timecourse was then calculated by dividing the difference between onset and peak magnitudes (e.g., rise) by the time between onset and peak (e.g., run).

Repeated-measures ANOVAs were conducted to determine if stimulus type and response speed were statistically significant predictors of the estimated slope of fMRI activity in face and house-selective regions. All pairwise comparisons were performed using a two-sided paired-samples t-test and were limited to hypothesis driven comparisons and only after the general effect was determined to be significant by a one- or two-way repeated measures ANOVA. Trials were sorted into fast and slow bins based on response times. The proportions of trials in each condition (stimulus [face, house] x cue validity [house, neutral, face] x response speed [fast, slow]) are plotted in Supp. Figure 1a. Mean response times for fast and slow face and house trials are plotted in Supp. Figure 1b. In line with past fMRI findings [1], we found a significant interaction between stimulus (face, house) and response speed (fast, slow) on the slope of increasing activation in face-selective regions (Supp. Figure 1c; $F(1,18)=9.22$, $p=.007$), with a steeper slope in face-evoked activity on trials with faster than slower "face" responses ($t(18) = 7.59$, $p<.0001$).

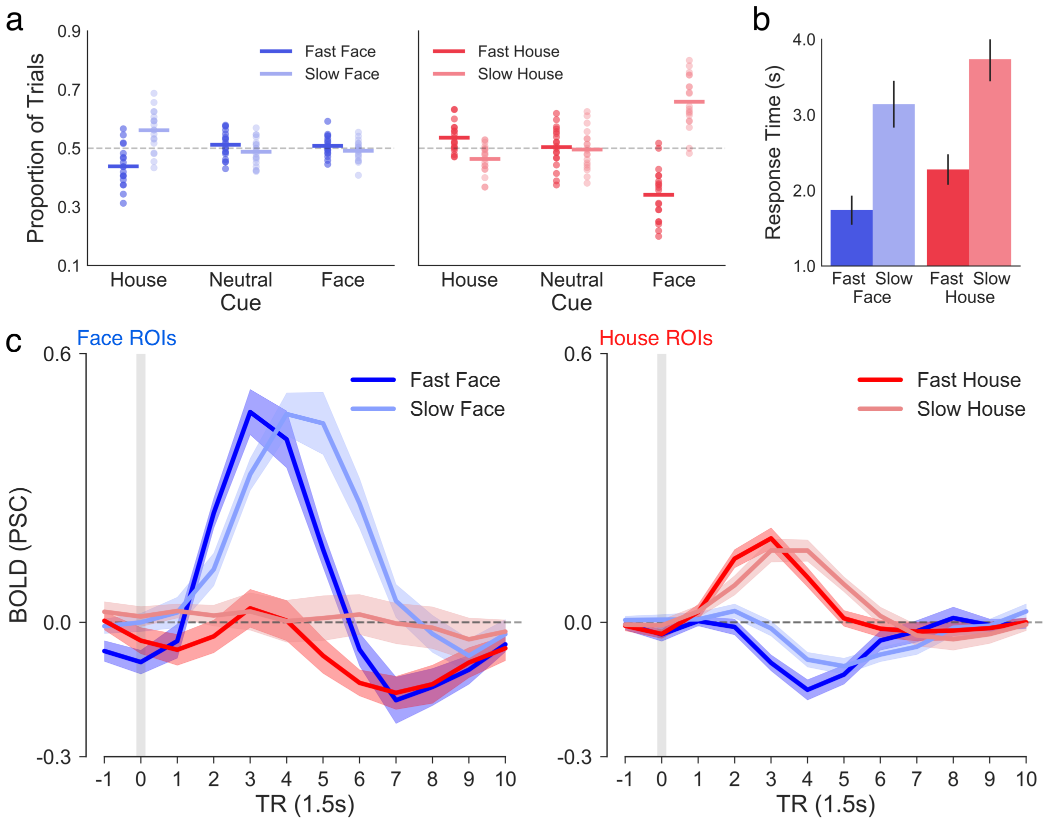

Supplementary Figure 1. Decision speed-dependent rise and onset of decay in category-selective ITC. **(a)** proportion of Fast (dark) and Slow (light) decisions within each cue condition for face (blue) and house (red) stimuli. Subject-averaged proportions are shown as horizontal lines with individual subjects shown by dots. **(b)** mean RT for Fast and Slow face and house decisions, plotted using the same color conventions as in **a. (d)** Face-selective ROIs displayed a steeper rising slope in activity for Fast compared to Slow face decisions. **(c)** In addition to showing a steeper slope for Fast compared to Slow house decisions, house-selective ROIs displayed a steeper negative deflection on Fast compared to Slow face decisions. Error bars in **b** reflects the 95% c.i. around the mean. Shaded area around activation timecourses in **c** and **d** reflects +/- 1 s.e.m.

With respect to the rising slope of activity in house-selective regions (Supp. Figure 1d), the interaction between stimulus type and response speed only reached marginal significance (F(1,18)=3.87, p=.07); however, both the main effects of stimulus (F(1, 18)=54.84, p<.0001) and response speed (F(1,18)=7.03, p=.016) reached statistical significance. Furthermore, similar to the effect of response speed on face-selective activity, a comparison of the slope of activity in house-selective regions on trials with fast and slow "house" responses revealed that faster

decision times were correlated with a significantly steeper slope in house-selective regions ($t(18) = 2.60$, $p=.018$). Due to the negative signal deflections observed in house-selective regions on face trials (Supp. Figure 1c, right), we also conducted an ANOVA to test for a significant interaction between stimulus type and response speed on the negative slope of activity. We found a significant interaction between stimulus type and response speed ($F(1, 18)= 12.03$, $p=.003$), confirming that fast "face" decisions were, indeed, associated with a steeper negative deflection of house-selective signals compared to slow face decisions ($t(18) = -3.57$, $p=.002$).

Individual differences in computational and neural measures of expectation bias. In addition to the group-level effects discussed in the main manuscript, we asked if individual differences in starting-point and drift-rate biases (see Supplementary Figure 2a) were correlated with subjects' observed levels of BOLD activity in ITC. It is important to note that analysis of individual differences between brain activity and behaviorally derived variables can lead to inflated [2] estimates of the covariance between neural and behavioral measures. Thus, we present these correlations simply to supplement the relationship established between group-averaged BOLD activity and group-level diffusion model parameters presented above. However, it is also important to point out that these correlations do not rely on circular inference, as individual model parameter estimates were not included in the regression models used to extract neural activity from subject-specific face- and house-selective ROIs, and diffusion model parameters were fit to the behavioral data only.

For each ITC region, the peak stimulus-evoked activation for the preferred category (e.g., peak face-evoked magnitudes in face-selective ITC) was extracted for each subject and regressed against subject-wise distance-to-threshold and drift-rate estimates using a linear mixed effects model with subject treated as a random effect (e.g., allowing separate intercepts

to be estimated per subject). Distance-to-threshold and drift-rate values were calculated for each subject by averaging across best-fitting values in each cue condition from the MSM. Each resulting vector of subject means was then converted into z-scores. In face-selective ROIs, we found distance-to-threshold ($\beta_{\Delta F}$ =0.274, *z*=2.301, *p*=0.02), but not drift-rate (β_{vF}= -0.288, *z*=-1.301, *p*=.19), to be a significant predictor of peak face activation across subjects (Supplementary Figure 2b, left). The positive relationship observed between subjects' estimated distance-to-threshold for face decisions and the magnitude of stimulus-evoked activity in face ROIs suggests that activity in this region, at least partially, reflects the required amount of evidence needed to make a face decision. The absence of a drift-rate effect on face-evoked activity may indicate a heavier reliance on expected than observed sensory evidence (see also Supplementary Figure 3), particularly when sensory evidence is scarce (as in the current task). However, another possibility is that, due to face decisions being relatively fast (~2 seconds), individual variation BOLD responses not be resolvable at the level required to detect covariation with behaviorally derived drift-rates.

In house-selective ROIs, both distance-to-threshold ($\beta_{\Delta H}$=-0.427, *z*=-2.832, *p*=.005) and drift-rate (β_{vH}=0.337, z=2.937, p=.003) reached statistical significance (Supplementary Figure 2b, right). In contrast to face-selective ROIs, house ROIs showed a negative relationship with distance-to-threshold estimates. One explanation for this effect is that subjects with a greater bias towards expecting faces (e.g., greater distance to the house threshold), may apply lower attentional gain to incoming house evidence, thus, evoking a weaker response in house-encoding sensory regions. The observed positive relationship between house drift-rate and the house-evoked activity seems to be consistent with this interpretation, as higher drift-rates imply stronger sensory gain and are predictive of greater levels of activity in house ROIs. However, future work will be required to more rigorously vet these interpretations.

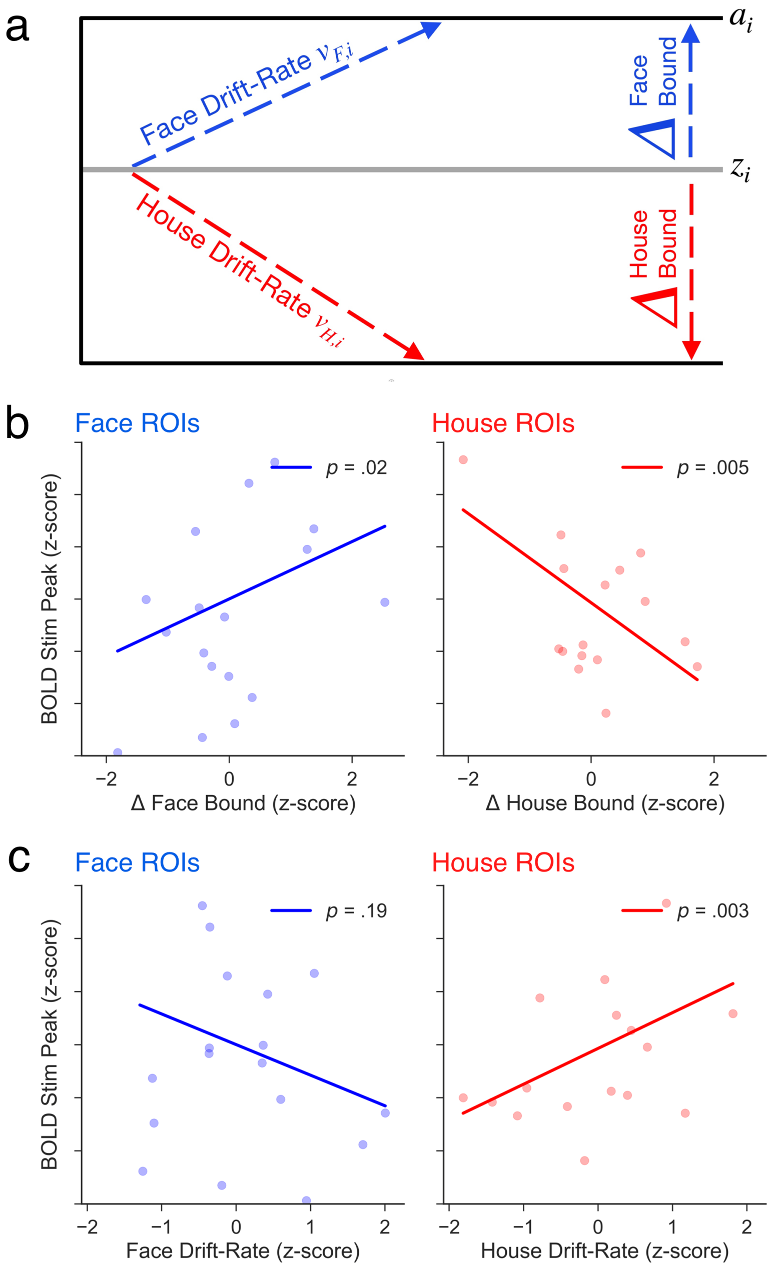

Supplementary Figure 2. Individual differences in DDM- and neural-based measures of expectation bias. **(a)** schematic of DDM showing the distance-to-threshold and drift-rate for a given subject. Vertical dotted lines show the distance-to-threshold for face (blue; top bound) and house (red; bottom bound). Peak face-evoked (left; blue) and house-evoked (right; red) BOLD magnitudes (e.g., estimated in the separated-events GLM) as a function of distance-to-threshold **(b)** and drift-rate **(c)** estimates on face and house trials. Lines show the simple slope estimated for each parameter on the x-axis. Regression analyses were performed using z-scored parameter estimates.

Estimating the relative contribution of prediction and error units to ITC activation. To assess the degree to which face and house-evoked activation in ITC reflects prediction and error signals, we fit the predictive coding model proposed in Egner et al., (2010) to peak BOLD magnitudes estimated by the combined-events GLM. The combined events GLM estimates the cue-locked timecourse of activity for each cue-stimulus pair, capturing the temporal sum of both prediction and stimulus-evoked activity, similar to the analysis performed in Egner et al., (2010). The model estimates the relative contributions of prediction (R) and error (E) units to the average peak response ($Y_{c,s}$) for each cue (C) and stimulus (S) pair as the weighted (w_X) sum, $\hat{Y}_{C,S} = w_E E_{C,S} + w_R R_{C,S}$. When fitting the model's two weight parameters to activity peaks in face-selective regions, R was set equal to the vector of cued face probabilities in each condition R=[.2, .5, .8, .2, .5, .8], reflecting expected face prediction magnitude in the following vector of conditions: [80H-Face, 50N-Face, 80F-Face, 80H- House, 50N- House, 80F-House]. For face stimuli, E was set equal to $1\text{-}R$ for all conditions including face stimuli, reflecting the degree of face "surprise". For house stimuli, E was set equal to 0, reflecting the assumption of PC that face-selective regions should not reflect error information for non-face stimuli (e.g., houses). Thus, in face-selective regions, E=[.8, .5, .2, 0, 0, 0]. The same conventions were used to define R and E vectors in house-selective regions, with values reflecting the strength of house predictions and prediction-errors that would be expected by PC.

In face-selective regions, we estimated a roughly 1.41:1 error to prediction weight ratio (w_E=.71, w_R=.51). This outcome reflects the same direction of bias toward error representation in face-selective ITC as that reported in Egner et al., (2010), albeit weaker in magnitude. Compared to the roughly 2:1 ratio observed in Egner et al., (2010), the smaller bias observed here is likely a reflection of differences in the stimuli used by Egner and colleagues, noiseless, grayscale face and house images, and those in our study, which were heavily noise distorted in order to increase the difficulty of the perceptual discrimination task, and to increase reliance on

prior cues. House-selective regions exhibited a comparatively stronger E:R bias, with house error and prediction representations contributing to the peak magnitude in house-selective ITC at about a 3.19:1 ratio (w_E=.30, w_R=.09).

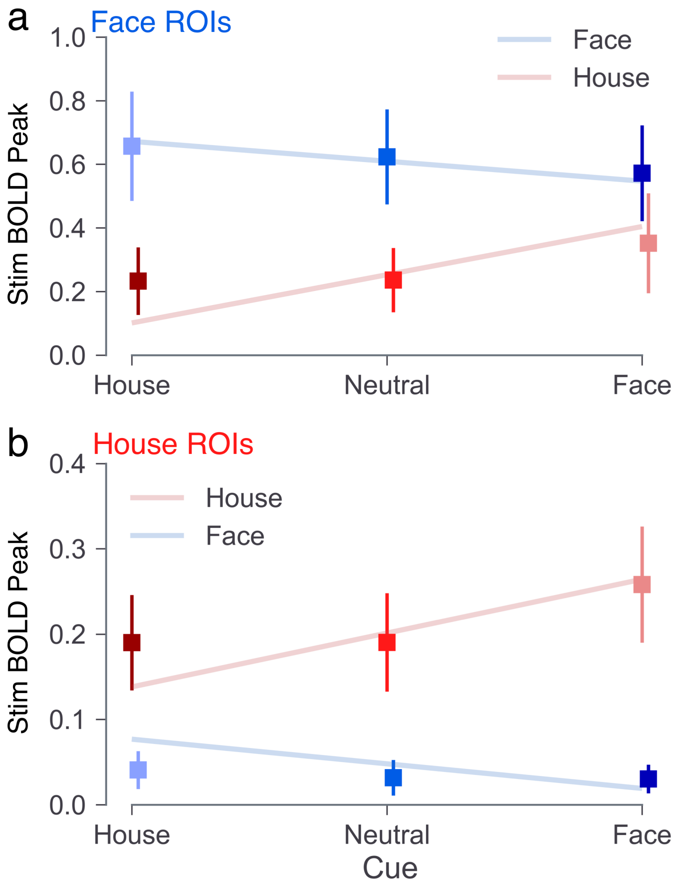

Supplementary Figure 3. Stimulus-evoked peaks (square markers) in **(a)** Face and **(b)** House ROIs in each cue condition estimated from the combined-events GLM with lines showing the simulated effect of expectation and surprise units for each stimulus category, based on fits of the predictive-coding model proposed by Egner and colleagues (2010).

Wholebrain analysis of pre- and post-sensory expectation effects. While the primary aim of the present study was to assess model-based hypotheses of pre- and post-sensory expectation biases in category-selective regions within ITC, it is undoubtedly true that ITC is only one node within a larger network of areas involved in computing perceptual and decision variables. Thus, we have provided a table (see Supplementary Table 1) listing wholebrain ROIs in which the

combined events GLM revealed a significant cue by stimulus by time interaction on full trials. Regions were identified by searching a smoothed image (4 mm FWHM) for voxels in which the cue, stimulus, time interaction exceeded a z-statistic threshold (-3.25 > Z > 3.25), with spherical ROIs grown with 12 mm radius around each identified peak. Voxels within the spherical ROIs that failed to pass a multiple comparisons and sphericity correction (*p*<0.05) were eliminated. To give readers further insight into the dynamics present in these ROIs, we also have included activation time-courses for eight of these ROIs (Supplementary Figure 4). The selected subset of regions for which activity timecourses are plotted in Supplementary Figure 4 were chosen on the basis of relevance to previous findings in the perceptual decision making literature and to highlight areas with the most interpretable cue-related modulation on catch and full trials.

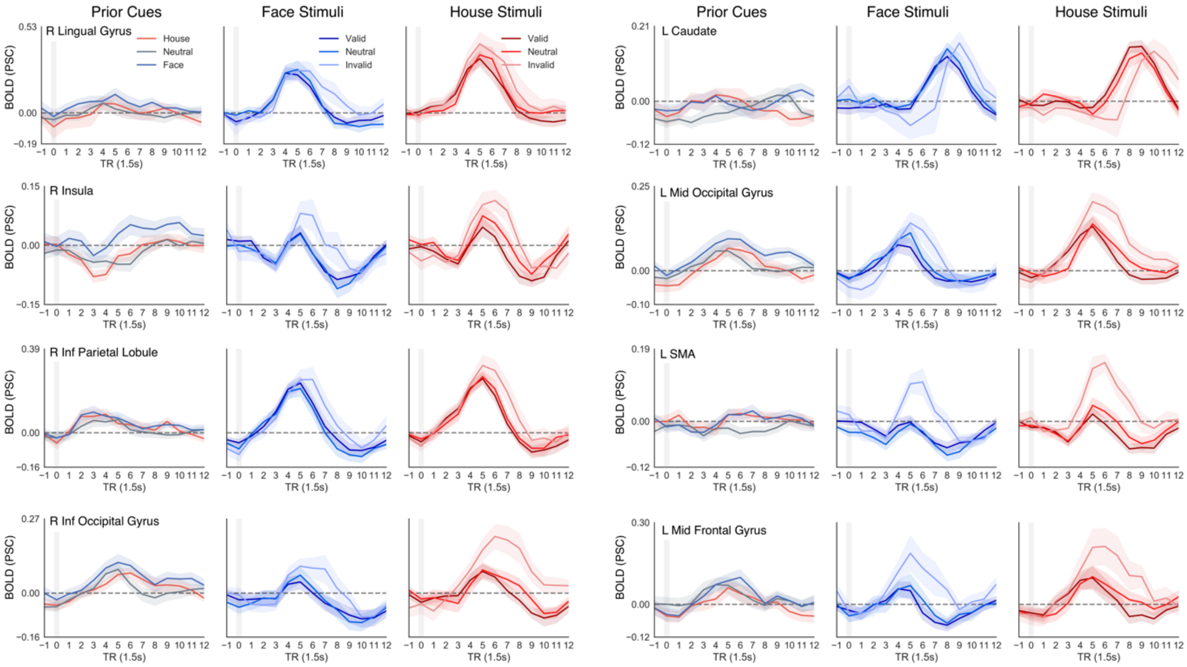

Supplementary Figure 4. Expectation effects on pre- and post-sensory activation throughout decision network (see Supplementary Table 1). Timecourse of the BOLD response to cues (left), face stimuli (middle, blue) and house stimuli (right, red) in select ROIs (ROI names in left axis of each panel) showing a significant interaction three-way interaction between cue, stimulus, and time in the combined-events wholebrain GLM analysis.

Supplementary Table 1. Wholebrain regions of interest (ROI) showing a significant interaction between cue, stimulus, and time in the combined-events GLM, including the number of voxels in each ROI (N_{vox}), and x, y, and z Talairach atlas coordinates. ROI abbreviations: L, left; R, right; Mid, middle; Inf, inferior; S Sulcus; Sup, superior; SMA, supplementary motor area.

ROI	N_{vox}	x	y	z
R Lingual Gyrus	51	17	-93	-10
R Postcentral Gyrus	87	23	-43	66
R Mid Frontal Gyrus	99	29	53	8
R Insula	279	31	19	-4
R Inf Occipital Gyrus	110	31	-89	-8
R Calcarine	90	3	-63	16
R Inf Parietal Lobule	524	43	-57	50
R Precentral Gyrus	774	47	9	38
R Sup Temporal Gyrus	39	65	-27	16
L SMA	737	1	19	52
L Pallidum	51	-13	41	0
L Caudate	101	-19	-3	26
L Precuneus	75	-19	-47	6
L Mid Occipital Gyrus	450	-29	-75	32
L Cerebellum	200	-33	-83	-30
L Inf Frontal Gyrus	36	-47	43	-2
L Mid Frontal Gyrus	135	-51	15	38
L Cuneus	36	-5	-71	36

References

1. Tremel, J. J. & Wheeler, M. E. Content-specific evidence accumulation in inferior temporal cortex during perceptual decision-making. *Neuroimage* **109,** 35–49 (2015).
2. Yarkoni, T. Big Correlations in Little Studies. *Perspect. Psychol. Sci.* **4,** 294–298 (2009).
3. Egner, T., Monti, J. M. & Summerfield, C. Expectation and surprise determine neural population responses in the ventral visual stream. *J. Neurosci.* **30,** 16601–8 (2010).
